# Supplementary material for: Identification of photoperiod-induced specific miRNAs in the adrenal glands of Sunite sheep (Ovis aries)
Source: Front Vet Sci. 2022 Jul 22;9:888207. doi: 10.3389/fvets.2022.888207 (PMC9354845; doi:10.3389/fvets.2022.888207)
Supplement: Supplementary file 1 [file Data_Sheet_1.ZIP › Supplementary file/miRDeep2 file.zip!/miRDeep2 file/Know_Structure/LP42A1/oar-mir-191.pdf]

miRBase precursor : oar-mir-191
Total read count : 117701
oar-miR-191 read count : 117350
remaining reads : 351

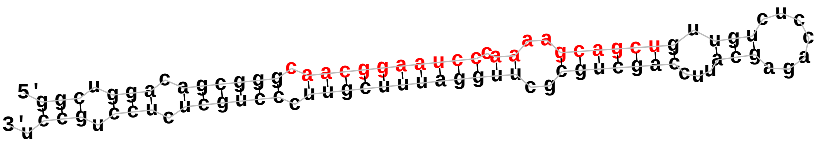

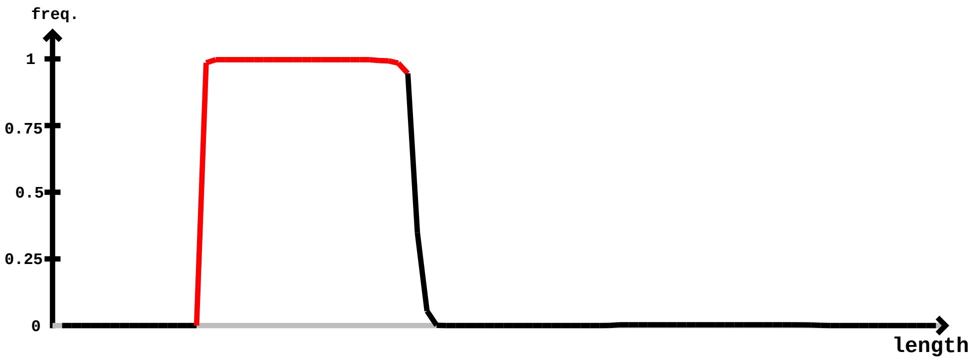

oar-miR-191

```
5'- ggcuggacagcgggcaacggaaucccaaaagcagcuguugucuccagagcauuccagcugcgcuuggauuucguucccugcucuccugccu  -3'    exp
    (((.(((.((((((.((((((((((.((..(((((((.(((.......)))...))))))).))))))))))))).)))))).))).))).    reads      mm         sample
    ..............caacggaaucccaaaag............................................................    1          0          seq
    ..............caacggaaucccaaaagc...........................................................    384        0          seq
    ..............caacggaaucccaaGagc...........................................................    1          1          seq
    ..............caacggaaucAcaaaagc...........................................................    1          1          seq
    ..............caacggaaucccaaaagca..........................................................    136        0          seq
    ..............caacggaaucccaaaagcag.........................................................    974        0          seq
    ..............caacggaaucccaaaagcagc........................................................    4551       0          seq
    ..............caacggaaucccaaaagcagcu.......................................................    69949      0          seq
    ..............caacggaaucccaaaagcagcug......................................................    33956      0          seq
    ..............caacggaaucccaaaagcagcugu.....................................................    5904       0          seq
    ..............caacggaaucccaaaagcagcuguu....................................................    122        0          seq
    ...............aacggaaucccaaaagc...........................................................    3          0          seq
    ...............aacggaaucccaaaagcag.........................................................    3          0          seq
    ...............aacggaaucccaaaagcagc........................................................    27         0          seq
    ...............aacggaaucccaaaagcagcu.......................................................    386        0          seq
    ...............aacggaaucccaaaagcagcug......................................................    553        0          seq
    ...............aacggaaucccaaaagcagcugu.....................................................    394        0          seq
    ...............aacggaaucccaaaagcagcuguu....................................................    1          0          seq
    ................acggaaucccaaaagcagcu.......................................................    3          0          seq
    ................acggaaucccaaaagcagcug......................................................    1          0          seq
    .......................................................agcugcgcuuggauuucgu.................    1          0          seq
    .......................................................agcugcgcuuggauuucguuc...............    2          0          seq
    ........................................................gcugcgcuuggauuucguu................    2          0          seq
    ........................................................gcugcgcuuggauuucguuc...............    16         0          seq
    ........................................................gcugcgcuuggauuucguucc..............    11         0          seq
    ........................................................gcugcgcuuggauuucguuccc.............    14         0          seq
    ........................................................gcugcgcuuggauuucguucccu............    5          0          seq
    .........................................................cugcgcuCggauuucguu................    1          1          seq
    .........................................................cugcgcuuggauuucguu................    4          0          seq
    .........................................................cugcgcuuggauuucguuc...............    13         0          seq
    .........................................................cugcgcuuggauuucguucc..............    31         0          seq
    .........................................................cugcgcuuggauuucguuccc.............    164        0          seq
    .........................................................cugcgcuuggauuucguucccu............    82         0          seq
    ..........................................................ugcgcuuggauuucguucccu............    5          0          seq
```
